# Epithelial production of elastase is increased in inflammatory bowel disease and causes mucosal inflammation

**DOI:** 10.1038/s41385-021-00375-w

**Published:** 2021-03-05

**Authors:** Jean-Paul Motta, Corinne Rolland, Anissa Edir, Ana-Carolina Florence, David Sagnat, Chrystelle Bonnart, Perrine Rousset, Laura Guiraud, Muriel Quaranta-Nicaise, Emmanuel Mas, Delphine Bonnet, Elena F. Verdu, Derek M. McKay, Etienne Buscail, Laurent Alric, Nathalie Vergnolle, Céline Deraison

**Affiliations:** 1 IRSD, Université de Toulouse, INSERM, INRAe, ENVT, UPS, Toulouse, France; 2 Unité de Gastroentérologie, Hépatologie, Nutrition, Diabétologie et Maladies Héréditaires du Métabolisme, Hôpital des Enfants, Toulouse, France; 3 Pole Digestif, CHU Toulouse, Toulouse, France; 4 Division of Gastroenterology, Department of Medicine, Farncombe Family Digestive Health Research Institute, McMaster University, Hamilton, ON Canada; 5 Department of Physiology and Pharmacology, University of Calgary, Calgary, AB Canada

## Abstract

Imbalance between proteases and their inhibitors plays a crucial role in the development of Inflammatory Bowel Diseases (IBD). Increased elastolytic activity is observed in the colon of patients suffering from IBD. Here, we aimed at identifying the players involved in elastolytic hyperactivity associated with IBD and their contribution to the disease. We revealed that epithelial cells are a major source of elastolytic activity in healthy human colonic tissues and this activity is greatly increased in IBD patients, both in diseased and distant sites of inflammation. This study identified a previously unrevealed production of elastase 2A (ELA2A) by colonic epithelial cells, which was enhanced in IBD patients. We demonstrated that ELA2A hyperactivity is sufficient to lead to a leaky epithelial barrier. Epithelial ELA2A hyperactivity also modified the cytokine gene expression profile with an increase of pro-inflammatory cytokine transcripts, while reducing the expression of pro-resolving and repair factor genes. ELA2A thus appears as a novel actor produced by intestinal epithelial cells, which can drive inflammation and loss of barrier function, two essentials pathophysiological hallmarks of IBD. Targeting ELA2A hyperactivity should thus be considered as a potential target for IBD treatment.

## Introduction

Inflammatory bowel diseases (IBD), which include Crohn’s disease (CD) and ulcerative colitis (UC), are chronic relapsing-remitting gut conditions, with an increasing worldwide incidence.^1^ Although the precise aetiology of IBD is still unknown, it involves a complex interaction between genetic, environmental, microbial factors, and immune responses.^2^ While most current treatments efficiently target the immune component of the disease, recent additional approaches also focus on restoring tissue architecture and functions.^3–6^ The goal of such new approaches is to delay and possibly avoid new flares, in an attempt to restore tissue homoeostasis. In the search for therapeutic targets that would meet those requirements, targets that regulate epithelial functions are of particular interest.^4–8^ Indeed, a functional epithelium warrants efficient barrier function, absorption, and controlled response to microbes.^5,6^ Proteases are among the mediators that are dysregulated in IBD tissues and that could affect epithelial functions.^9–11^ A number of studies have demonstrated that serine protease activity was significantly increased in animal models of colitis and in patients suffering from IBD.^12–16^ In particular, elastase activity was largely detected in mucosal tissues from IBD patients, even at sites distant from the inflammation (tissues with no inflammatory signs).^13^ Inhibition of general elastase activity by delivery of the protease inhibitor ELAFIN had protective effects in rodent models of colitis.^12,13^ Taken together, those findings support the concept that the elastolytic balance (protease/anti-protease activities) in IBD is broken, and that proteases from the elastase family take on a key role in the pathogenesis of intestinal inflammation. In particular, epithelial functions might be severely flawed by such elastolytic overload. Aims of the present study were to identify the elastolytic protease(s) expressed and active in tissues from IBD patients and to understand the role of such elastolytic activity in epithelial cell biology.

Using in situ zymography in tissues from IBD patients, we observed that most elastolytic activity was originating from the epithelium. We identified elastase 2A (ELA2A) as the elastase protease expressed and secreted by human colonic epithelial cells. We further deciphered the contribution of this form of epithelial elastase in key pathways associated with IBD pathophysiology.

## Results

### Elastase activity is detected in human intestinal epithelial cells (IEC)

**Fig. 1:**
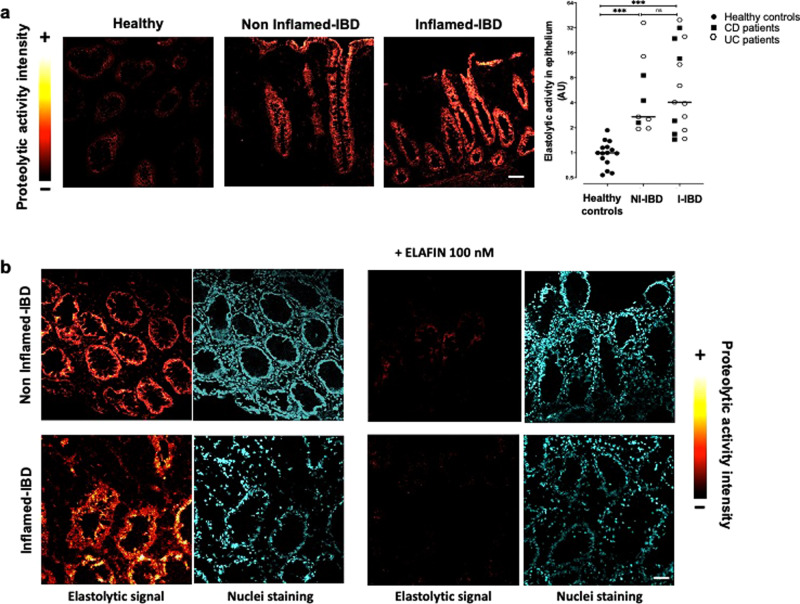
Elastolytic activity is increased in epithelial cells from IBD colonic biopsies. **a** In situ zymography of elastolytic activity in colonic mucosal biopsies from control groups (healthy) and inflammatory bowel disease (IBD) patients taken in non-inflamed and inflamed areas. Images colouring correspond to the intensity of fluorescence released after the proteolytic cleavage of elastine substrate according to the reported scale. Representative images of healthy controls tissue section (*n* = 15), non-inflammatory (NI-IBD) (*n* = 9), and inflammatory (I-IBD) areas from IBD patients (*n* = 15). Medians are represented. ***p < 0.001 compared to healthy patients using two-way ANOVA with Bonferroni post-test. Scale bar = 20 μm. **b** In situ zymography of elastolytic activity in colonic mucosal biopsies in presence or not of 100 nM of ELAFIN, an elastase inhibitor, added exogenously to the tissue slices. These pictures are representative images generated in five patients suffering from CD and UC and healthy control patients. DAPI staining showing nuclei is also represented for each section. Scale bar = 50 μm.

We investigated the cellular source of elastolytic activity in human colons from IBD patients and controls, using in situ zymography. Elastase activity in colon tissues from healthy individuals was weak, and was only detected in the epithelium (Fig. 1a). In colonic tissue from IBD patients (CD patient shown in Fig. 1a), elastolytic activity was higher than what was detected in tissues from healthy controls (Fig. 1a). This increased elastolytic activity was detected in IBD tissues from both inflamed and also non-inflamed areas (Fig. 1a, b). In inflamed areas of IBD patient tissues, some elastolytic activity was detected in the submucosa, although the strongest activity was localized in epithelial cells. Quantification of elastolytic activity signal in epithelium revealed that similar increase was observed in non-inflamed and inflamed IBD tissues, compared to healthy controls (×8.4 in non-inflamed area and ×11.46 in inflamed area from colonic biopsies of IBD patients compared to healthy controls) (Fig. 1a, b). To confirm the detection of elastase activity in human colon tissues, ELAFIN, an inhibitor specific for elastase activity, was applied on tissue sections. ELAFIN incubation completely inhibited tissue elastolytic activity in IBD patients (Fig. 1b). These data illustrated that epithelial cells produced elastase activity, which is upregulated in IBD conditions.

### Forms of elastase expressed in IEC

**Fig. 2:**
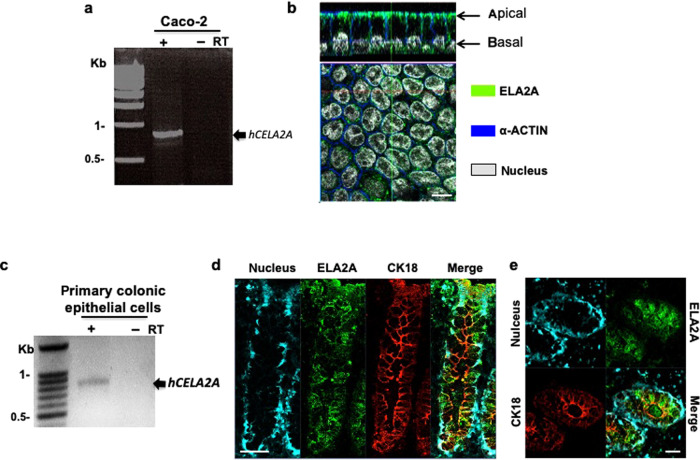
ELA2A is expressed and secreted by intestinal epithelial cells. **a** Human *CELA2A* cDNA was amplified from Caco2 transcripts after RT-PCR experiment. **b** ELA2A immunostaining on polarized Caco-2 cells cultured on transwells. Orthogonal view of Caco-2 cell monolayer showing ELA2A staining in green and ACTIN network in blue and nuclei in grey. This picture is representative of three independent experiments. Scale bar = 10 µm. **c** Human *CELA2A* transcript was amplified from isolated colonic crypt from healthy biopsy after RT-PCR experiment. ELA2A immunostaining on longitudinal section (**d**) and on Z cross-section (**e**) of healthy area from human colonic resection. Pictures are representative images of more than nine slices acquired from each individual. Signal of ELA2A is in green and signal related to epithelial cell marker, cytokeratin 18 (CK18), is in red. Nuclei are detected by DAPI staining and appear in blue. **d** Scale bar = 50 μm. **e** Scale bar = 25 μm.

To determine the identity of the elastase form(s) expressed in IEC, RT-PCR was performed with mRNA from Caco-2 cells, a human IEC line, using primer pairs targeting the five known human elastase genes (neutrophil elastase: ELANE, members of the chymotrypsin-like elastase family: *CELA1*, 1, *CELA2*, and *CELA3*, and matrix metalloproteinase-12: *MMP12*). RT-PCR analysis revealed the presence of only one enzyme transcript in IEC (Fig. 2a and Supplementary Fig. 1a–f). Sequencing of the PCR product revealed 100% identity with the full-length cDNA from *CELA2A* gene (NCBI accession number NM_033440; Swiss-Prot accession number P08217, ELA2A as protein name).

ELA2A protein expression was then confirmed in IEC line Caco-2 and in human colonic tissues (Fig. 2a–e). Analysis of ELA2A localization showed that in IEC monolayers, ELA2A signal was concentrated at the membrane level. Transversal view of cells evidenced that ELA2A immunoreactivity was closely associated with the plasma membrane, both at the apical and basolateral sides (Fig. 2b).

We confirmed *CELA2A* expression in isolated human colon crypts (Fig. 2c) and also showed transcriptional expression of its orthologous *Cela2A* in murine colon (Supplementary Fig. 1e). In human colonic tissues, immunostaining signal corresponding to ELA2A protein was observed along the colonic crypt, suggesting that ELA2A is produced by all colonic epithelial cell types (Fig. 2d). On cross-sections, staining was observed in the lumen, supporting the fact that IEC can secrete ELA2A (Fig. 2e), which is concordant with metaproteomic study from Li et al.^17^

### Control and regulation of ELA2A in intestinal epithelium

As previously described in the skin,^18^ ELA2A activity can be tightly controlled by ELAFIN, an elastase inhibitor (Supplementary Fig. 2a). To assess possible interactions between ELA2A and ELAFIN in colonic tissues, double-labelling immunofluorescence experiments were performed. ELAFIN staining appeared as dots in cytoplasm and also concentrated at the plasma membrane (Supplementary Fig. 2b). ELA2A and ELAFIN immunoreactivity overlapped in plasma membrane, cytoplasm, as well as in the lumen. These data thus support possible interactions between the ELA2 protease and its inhibitor ELAFIN in the colonic mucosa.

Proteolytic activity is also regulated by the pro-peptide cleavage of inactive zymogen forms of proteases. A previous study has demonstrated that ELA2A exists as a pro-form, and that pro-ELA2A could be converted into an active form, by trypsin cleavage.^18^ As colonic epithelial cells expressed three different forms of TRYPSINS (PRSS1, PRSS2, and PRSS3),^19^ we tested the effect of trypsins on pro-ELA2A activation. Co-incubation of pro-ELA2A with  TRYPSINS led to increased elastolytic activity compared to activity of pro-ELA2A basal activity (Supplementary Fig. 2c). PRSS1 and PRSS2 were more efficient (up to sevenfold increase) than PRSS3 at converting pro-ELA2A into active elastase. These data suggest that ELA2 activity could be regulated by epithelial trypsins.

**Fig. 3:**
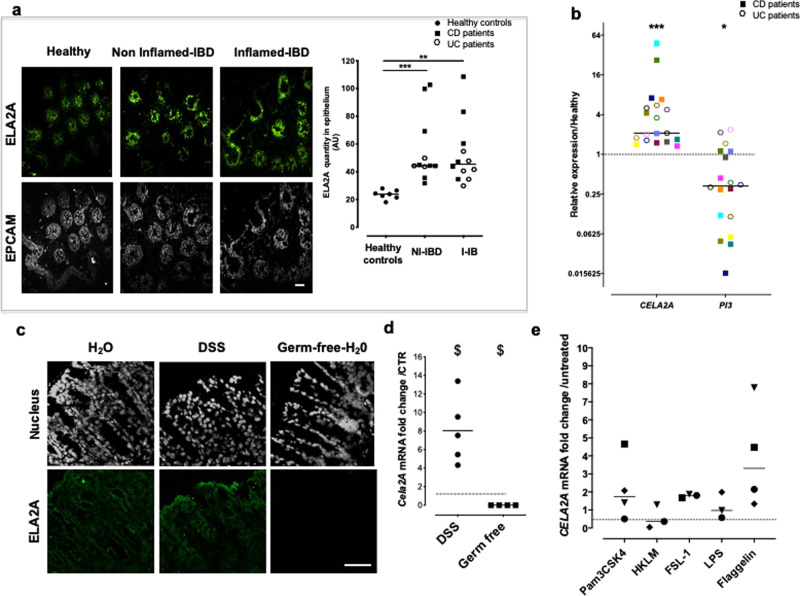
IBD-associated ELA2A epithelial overexpression. **a** ELA2A immunostaining on cross-section of colonic biopsy from healthy controls, non-inflamed and inflamed areas from IBD patient tissues using anti-ELA2 (green) and anti-EpCAM (Epithelial cell adhesion molecule, grey) antibodies, the latter identifying the epithelial compartment. Scale bar = 50 µm. Quantification of ELA2A signal in healthy controls (*n* = 5) versus non-inflammatory (NI-IBD) (*n* = 11) and inflammatory (I-IBD) areas from IBD patients (*n* = 12). Data represent the intensity of ELA2A signal per unit of epithelial surface. Medians are represented. **p < 0.01, ***p < 0.001 compared to healthy patients using two-way ANOVA with Bonferroni post-test. **b** Gene expression of *CELA2A* and *PI3*, encoding ELA2 and ELAFIN, respectively, in isolated epithelial colonic crypts. Data represent fold change expression compared to healthy controls. Patient are individualized by different colours, CD patients are represented by filled square (*n* = 11) and UC patients by an open hexagone (*n* = 8). Medians are represented. ***p < 0.001, *p < 0.05 compared to healthy patients using a Mann–Whitney’s non-parametric *t*-test. **c** ELA2A immunostaining in colon from naive mice, mice with DSS-induced colitis or germ-free mice. Scale bar = 50 µm. Images are representative of at least three mice in each group. **d** *Cela2a* mRNA quantification in colonic tissues from colitis (*n* = 5) and germ-free (*n* = 4) mice. ^$^*p* < 0.05 compared to control mice (dashed line) using a Mann–Whitney’s non-parametric *t*-test. **e** Gene expression of CELA2A in human colon organoid after stimulation with Toll-like receptors agonists (Pam(3)CSK(4): TLR2/TLR1, HKLM: TLR2, FSL-1: TLR2/TLR6, LPS: TLR4, and flagellin: TLR5).

In the colonic mucosa of IBD patients, ELA2A immunostaining was more intense compared to its expression in healthy control tissues (Fig. 3a). Higher ELA2A expression was detected in both inflamed and non-inflamed IBD tissues (Fig. 3a). This finding correlates to what was observed for elastolytic activity detected by in situ zymography (Fig. 1a). Quantitative analysis showed a twofold increase for ELA2A expression in IBD tissues, compared to tissues from healthy controls (Fig. 3a right panel). This significant increase was in the same order of magnitude in both inflamed (×2.16 ± 0.34) and non-inflamed mucosa (×2.41 ± 0.34) and was similar in CD and UC patients (×2.6 and ×2-fold increase, respectively, compared to healthy controls). Therefore, the increased expression of ELA2A seemed to be independent of the macroscopic inflammatory status of the colon in IBD patients, but is clearly associated with IBD. The transcriptomic analysis of epithelial cells isolated from non-inflamed colon of IBD patients confirmed that *CELA2*A mRNA amount was increased by more than sixfold in IBD epithelium, compared to epithelial cells from control patients (Fig. 3b). The *CELA2A* gene expression increased by ninefold in epithelial cells from colonic CD tissues and by 3.3-fold in UC tissues. In the IBD epithelial isolates, gene encoding ELAFIN, *PI3*, was downregulated (53%), compared to healthy tissues (Fig. 3b).

To further evaluate the regulation of ELA2A expression during inflammation, ELA2A immunostaining was performed on colonic slices from mice submitted to DSS colitis (5%; for 7 days). In colonic tissues from control or DSS-induced colitis mice, ELA2A immunoreactivity was strong in epithelial cells and during inflammation epithelial ELA2A staining was higher than in control group (Fig. 3c). Transcription level of *Cela2A* mRNA was higher in colitis condition compared to control mice (Fig. 3d). Interestingly, *Cela2a* expression was absent in colonic tissues from germ-free mice (Fig. 3c, d). We used human colonic primary epithelial cells to highlight which environmental stimuli could drive *CELA2A* upregulation. Pro-inflammatory cytokines cocktail (IL6, TNFα, IL1β) added to culture media of human colonic organoids had no effect on *CELA2A* gene transcription (Supplementary Fig. 3a). Toll-like receptors stimulation was also investigated (Fig. 3e) and flagellin, a bacterial motif recognized by TLR5, seemed to induce an upregulation of ELA2A encoding mRNA in stimulated human colonic epithelial cells. In contrast, TLR activation did not induce expression change of gene encoding direct ELA2 inhibitor, the ELAFIN (Supplementary Fig. 3b).

### In vivo ELA2A upregulation causes mucosal inflammation

**Fig. 4:**
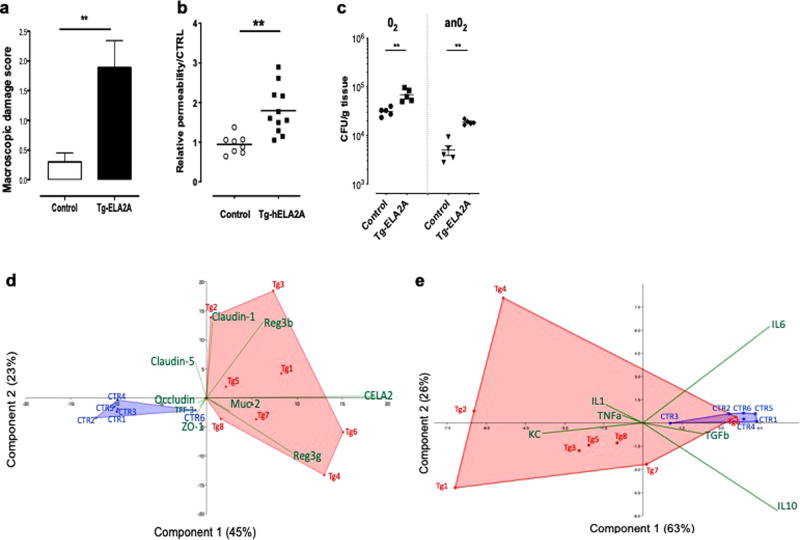
In vivo overexpression of hELA2A in mouse intestinal epithelial cells leads to a pro-inflammatory phenotype. **a** Macroscopic damage scores of colon from ELA2A transgenic mice and control after 30 days of tamoxifen injections. Data are means ± SEM. **p < 0.01 by two tailed *t*-test with Mann–Whitney post-test. **b** In vivo intestinal permeability measured by FITC–dextran concentration in blood from control (*n* = 8) and hELA2A overexpressing mice (*n* = 11). **c** Anaerobic (AnO_2_) and aerobic (O_2_) bacterial translocation to mesenteric lymph nodes (*n* = 5 per group), as measured by bacteria colony counting in Columbia blood agar plates**. d** Transcriptomic analysis of genes related to epithelial barrier function in mucosae from Tg-ELA2A and controls mice represented as principal component analysis (Euclidian matrix) illustrating significant shift of Tg-ELA2A transcriptome (Tg in red) compared to control littermate (CTR in blue) (PERMANOVA Bonferroni *p* = 0.0002). Biplot vectors (green) indicate the maximum increase and strength of each variable to the overall distribution. The first two principal axes explained 68% of the variance. **e** Principal component analysis (Euclidian matrix) of transcriptional variation of genes related to inflammation in colonic tissue from Tg-ELA2A and controls mice revealed significant shift of Tg-ELA2A transcriptome (Tg in red) compared to control littermate (CTR in blue) (PERMANOVA Bonferroni *p* = 0.003). Biplot vectors (green) indicate the maximum increase and strength of each variable to the overall distribution. The first two principal axes explained 89% of the variance.

In vivo pathophysiological consequences of ELA2A hyperactivity in IEC were studied in transgenic mice expressing human ELA2A, specifically in IEC (see transgene construction in Supplementary Fig. 4a). Western blot and immunohistochemistry analysis confirmed ELA2A overexpression in murine colonic epithelium (Supplementary Fig. 4b,c). Increased elastolytic activity was released in luminal washes of hELA2A transgenic mice compared to controls (Supplementary Fig. 4d). In situ zymography showed a more intense epithelial elastolytic activity in hELA2A transgenic mice compared to controls (Supplementary Fig. 4e). Chronic ELA2A hyper-activity (30 days) induced macroscopic damage in the colon (Fig. 4a), a disrupted intestinal barrier and increased bacterial translocation to mesenteric lymph nodes (Fig. 4b, c), as well as upregulation of pro-inflammatory genes (Fig. 4e). Histological analysis revealed damage of the mucosa where the epithelium was severely affected (sites of erosion), infiltrated mononuclear cells, macrophages, and vasodilation (Supplementary Fig. 5a, b). In addition, in hELA2A transgenic mice, goblet cell depletion was observed (Supplementary Fig. 5a, c).

**Fig. 5:**
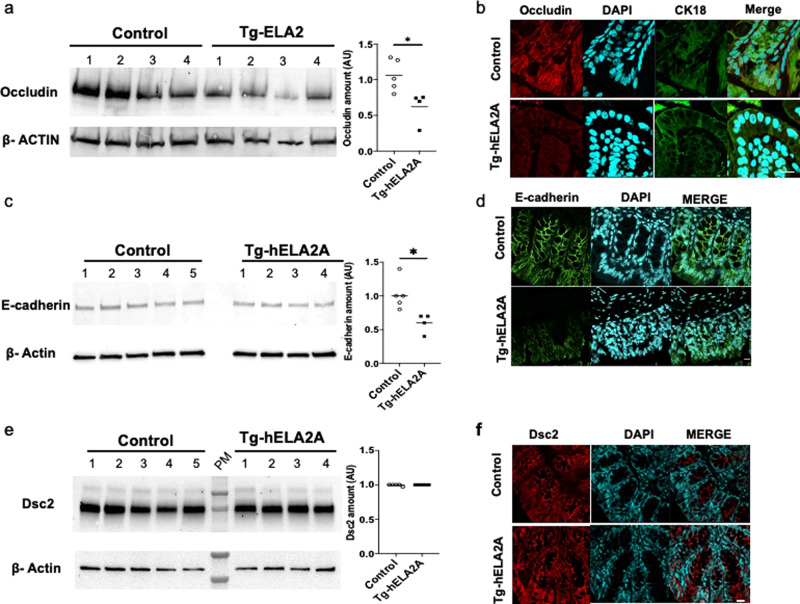
ELA2A hyperactivity cleaves proteins involved in cell–cell junctions. Protein level of tight junction protein occludin (**a**), adherent junction protein E-cadherin (**c**), and Desmocollin-2 involved in desmosomes (**e**) were assessed by western blot analysis in protein extraction from control and Tg-ELA2A colonic mucosae (*n* = 4 per group). Quantification analysis was performed using β-actin as loading reference and means are represented. *p < 0.05 by two tailed *t*-test with Mann–Whitney post-test. Immunostaining of the tight junction protein Occludin and the epithelial cell marker CK18 (**b**), E-cadherin (**d**), and Desmocollin-2 (**f**) in colonic tissue sections of control and hELA2A overexpressing mice. Presented pictures are representative of staining obtained with five mice per group. Nuclei are detected by DAPI staining and appear in blue. **b** Scale bar = 10 μm. **d**, **f** Scale bar = 20 μm.

Concomitantly with loss of barrier function, immunodetection of occludin was lower at the membrane level in hELA2A transgenic mice compared to controls (Fig. 5a, b), suggesting that ELA2A overexpression caused disrupted barrier function, potentially through proteolytic degradation of such important tight junction-associated protein. Quantification of Occludin amount by western blot confirmed a decrease of this protein essential in tight junction’s ability to seal the paracellular space in hELA2A transgenic mucosa compared to controls (*p* < 0.05) (Fig. 5a).

In addition, examination of E-cadherin, a major component of adherent junction, showed that this intercellular protein was also a target of ELA2 hyper-activity (Fig. 5c, d). Western blot and immunostaining analysis revealed a decreased amount of E-cadherin, whereas no transcriptional variation was observed in hELA2A transgenic mucosa (Supplementary Fig. 6).

In contrast, pattern distribution of Desmocollin-2, a trans-membrane protein involved in desmosomes, was not impacted by elastolytic hyper-activity in transgenic animals (Fig. 5e, f), suggesting specific substrates for ELA2 proteolytic activity.

Moreover, the transcriptomic analysis revealed a clear difference between the colonic mucosa from hELA2A transgenic mice and controls visualized by principal component analysis (PCA) (PERMANOVA *p* value = 0.0002). This analysis indicated the contribution of dysregulation of genes encoding *Reg3g, Reg3b, Muc2*, and trefoil factor-3 (*TFF-3*) in mucosa (Fig. 4d). In whole tissue, PCA analysis segregated both groups and the strongest discriminants were the transcriptional variations of *KC*, *IL-1, IL-6*, *IL-10*, and *TGFβ* genes (PERMANOVA *p* value = 0.0002) (Fig. 4e). Transcript amounts of *Reg3g, Reg3b, Muc2, KC*, and *IL-1* genes were all significantly increased compared to controls. Gene expression of mediators involved in epithelial protection and repair such as *TFF-3*, *IL-6*, *IL-10*, and *TGFβ* was downregulated in tissues from ELA2A transgenic mice, compared to controls (Supplementary Fig. 6b). Altogether, these data demonstrated a pro-inflammatory phenotype associated with the in vivo overexpression of human ELA2A in IEC. This phenotype associates intestinal barrier defects and overproduction of epithelial defence factors, at the expense of resolution and repair signals.

### Mechanisms involved in ELA2A-induced barrier function defects and cytokine dysregulation in human IEC

**Fig. 6:**
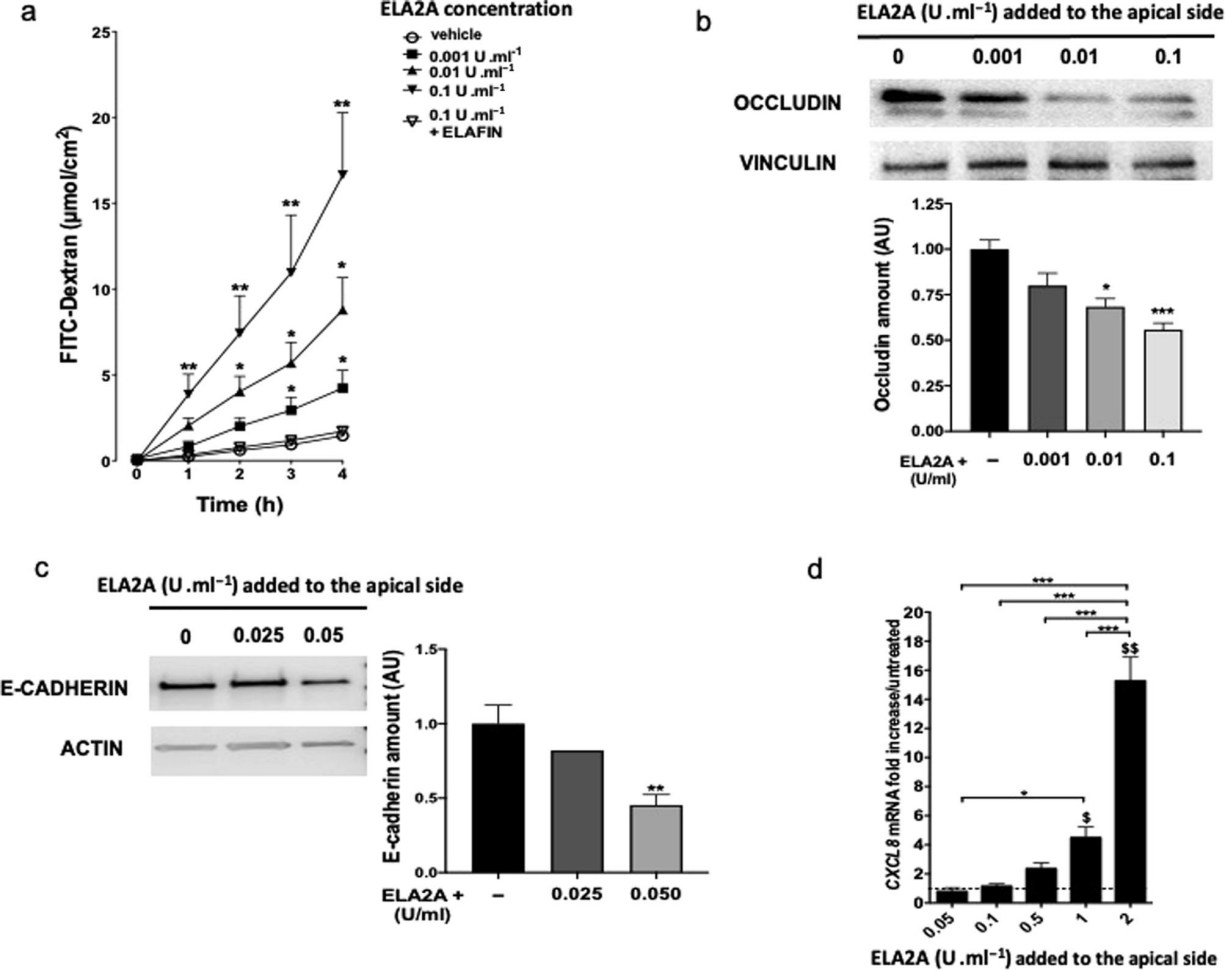
Increased extracellular ELA2A in Caco-2 cells alters the epithelial barrier function. **a** FITC–dextran flux after incubation of recombinant human ELA2A on the apical side of polarized Caco-2 monolayer. ELA2A, preincubated or not with an elastase inhibitor ELAFIN (100 nM), was added to the apical side of Caco-2 cells for 4 h. Data are the means of three independent experiments with *n* = 3 per group. *p < 0.05, **p < 0.01 compared to control condition using two-way ANOVA test with Bonferroni post-test. **b** OCCLUDIN amount in membrane fraction of polarized Caco-2 monolayer after addition of various concentrations of recombinant ELA2A onto the apical medium. VINCULLIN was used as a loading control for western blot analysis. Blots are representative of three independent experiments. Quantification analysis was performed and data are means ± SEM of three experiments with *n* = 2 per group; **p < 0.01; ***p < 0.001 by one-way ANOVA with Bonferroni post-test. **c** E-cadherin amount in total fraction of polarized Caco-2 monolayer after addition of various concentrations of recombinant ELA2A in the apical medium for 4 h. ACTIN was used as a loading control for western blot analysis. Blots are representative of at least three independent experiments. Quantification analysis was performed and data are means ± SEM; **p < 0.01; ***p < 0.001 by one-way ANOVA with Bonferroni post-test **d** Quantification of *CXCL8* transcripts after stimulation of Caco-2 monolayers by different concentrations of recombinant ELA2A. Data are expressed as means ± SEM of *n* = 3 for each group in three independent experiments, *p < 0.05, **p < 0.01 compared to control, ^$^*p* < 0.05 and ^$$$^*p* < 0.001 for all comparisons indicated by the bracket using two-way ANOVA with Bonferroni post-test, dashed line corresponds to mRNA level in control cells.

To determine the direct role of ELA2A in IEC, we exposed Caco-2 monolayers to exogenous purified ELA2A and we analyzed barrier function after 4 h. Apically applied ELA2A induced a significant concentration-dependent increase of dextran apical to basolateral flux (*p* < 0.05), as well as a decrease of trans-epithelial electrical resistance (*p* < 0.01) (Fig. 6a and Supplementary Fig. 9b). Pre-incubation of ELA2A with the elastase inhibitor ELAFIN prevented this loss of barrier function (Fig. 6a), demonstrating that ELA2A elastolytic activity was responsible for increased permeability. As measured by western blot, amounts of occludin protein in membrane fractions were also significantly reduced by exposure to exogenous ELA2A (*p* < 0.001) (Fig. 6b) despite no transcriptional change (data not shown). Addition of ELA2A to the apical side of epithelial layer also induced the degradation of E-cadherin protein in a dose-dependent manner (Fig. 6c).

The defect of barrier function was maintained after long-term exposure of hyperactive ELA2A. This is evidenced by the greatly enhanced flux of macromolecule and *E. Coli* bacteria from the apical to the basolateral side of monolayer of ELA2A-overexpressing Caco2, compared to control cells (Supplementary Fig. 7a–c). Addition of ELAFIN to the extra-cellular medium showed that ELA2A-induced loss of epithelial barrier function was dependent on elastolytic activity (Supplementary Fig. 7b). These characteristics were confirmed in another human intestinal epithelial cell line, HT-29 (Supplementary Fig. 8).

Next, we studied the involvement of protease-activated receptors (PARs), as possible mechanisms of ELA2A-induced increased epithelial permeability. We used specific antagonists of PAR1 or PAR2 (F16357 and GB88, respectively), both known to increase epithelial permeability. None of these two antagonists blocked the decrease of trans-epithelial resistance or the increased macromolecule flux (4 kDa dextran) induced by ELA2A proteolytic activity (Supplementary Fig. 9a–c).

We also analyzed cytokine profile in epithelial cells exposed to purified ELA2A. Four hours after the exogenous addition of ELA2A to the apical side of Caco-2 monolayers, *CXCL8* mRNA was upregulated (*p* < 0.001) in a concentration-dependent manner (Fig. 6d). This upregulation of *CXCL8* transcription was sustained after a chronic exposure of hyperactive ELA2 (in cells overexpressing ELA2A) and was also accompanied by decrease of *IL-10* and *TGFb* expression (Supplementary Figs. 7d and 8c). Treatment of epithelial cells with chymostatin, a small chemical ELA2A inhibitor, decreased in a concentration-dependent manner the CXCL8 release in extra-cellular medium of ELA2A-overexpressing Caco2. Hence, demonstrating that CXCL8 secretion is dependent on the proteolytic activity of ELA2 (Supplementary Fig. 7e). Treatment with PAR2 (GB88) or TRPV4 (5HC067064) antagonists prior to ELA2A addition to Caco2 monolayers did not reduce the *CXCL8* upregulation (Supplementary Fig. 9d). In contrast, PAR1 antagonist (F16357) or NFκB inhibitor (BAY 11-7082) treatment drastically decreased the ELA2 capacity to induce *CXCL8* upregulation. These results demonstrate that ELA2A regulates *CXCL8* expression by a mechanism involving both PAR1 and NF-κB activation (Supplementary Fig. 9d).

## Discussion

In this study, we demonstrate that IEC produce and secrete an active form of elastase, ELA2A. Despite sharing the same generic name with neutrophil elastase (ELANE), this elastase is very different (only 26% amino acid identity). ELA2A has different substrate affinity from ELANE, as it is more closely related to enzymatic action of chymase (chymotrypsin-like activity). Therefore, we hypothesized that these two enzymes have different functions and specificity, and that particularly in the context of inflammation, they might have different substrates, and different roles.

We showed that ELA2A is constitutively expressed and released by colonic epithelium from mouse and human. In addition, we highlighted ELA2A upregulation in intestinal epithelium from patients suffering from CD and UC. We demonstrate that epithelial ELA2A hyperactivity leads to a loss of intestinal barrier function, a decrease of anti-inflammatory factors, and an increase in pro-inflammatory epithelial mediators. Altogether, this supports a role for this protease in epithelial pathophysiology and in chronic inflammation pathways.

In recent years, there has been enormous progress in understanding the pathogenesis of CD and UC. The importance of the mucosal barrier in preventing bacterial invasion and subsequent inflammation has been shown in genetic studies identifying risk genes and in animal models.^20–22^ Increased epithelial permeability can play a central role in inflammatory processes, where impaired permeability often reflects a primary defect of the epithelial tight junctions.^23,24^ Trying to restore epithelial barrier function is therefore a tempting therapeutic option. However, defining the mediators that are the most important in initiating epithelial barrier’s dysfunction is the first step to this approach.^25^

We find that epithelium represents the major source of elastase activity in endoscopically inflamed areas of IBD colon, as well as in non-inflamed areas. This suggests that increase of epithelial elastolytic activity is associated with IBD regardless of the endoscopic inflammatory status of the tissues. Increased epithelial elastase activity could therefore be among the dysregulated epithelial factors that could initiate barrier dysfunction associated with IBD.

The ELA2A quantity is increased in IBD epithelium independently of the inflammatory status of colonic tissues, compared to healthy tissues. This was associated with elevated elastolytic activity measured in culture supernatants of biopsies harvested from both inflamed and non-inflamed areas of the colon of IBD patients, compared to supernatants of biopsies from healthy controls.^13^ *Cela2a* upregulation (twofold increase, *p* < 0.05) was observed in different models of colitis such as acute DSS-induced colitis (analysis of GEO data base number GSE5864^26^) or chronic colitis induced by TNBS administration (analysis of GEO data base number GSE35609^27^). These results are in accordance with the increased elastolytic activity we observed in epithelium from mouse model of colitis.^13^ IEC also express ELAFIN, which is a potent ELA2A inhibitor.^18^ Co-immunolocalisation of ELA2A and ELAFIN shows that both proteins are secreted together in the colonic lumen. Consistently with our previous observation,^13^ we showed in the present study that concomitantly with *CELA2A* upregulation, ELAFIN expression is abolished in epithelial cells. Therefore, the absence of endogenous inhibitor would allow ELA2A to participate to the elevated elastolytic activity released by IBD biopsies. Therapeutic intervention based on ELAFIN delivery in colonic lumen drastically reduced the epithelial elastolytic activity^13^ and could therefore potentially reverse the inflammatory effects of ELA2A hyper-activity.

Considering the upregulation of ELA2A expression in IBD condition, we investigated potential stimuli that could regulate its expression in IEC. Colitis condition led to increase of ELA2A amount into epithelial cells. Using human colonic organoids, we found that IL6, TNFα, IL1β, or TLR1, 2, 3, 4, and 6 agonists added to culture media had no effect on *CELA2A* gene transcription. However, bacterial component could influence *CELA2A* transcriptional regulation. Indeed, ELA2A is absent in germ-free epithelium in accordance with proteomic approach showing large decrease of ELA2A amount in germfree mice.^28^ TLR5 stimulation could be involved in *CELA2* upregulation observed in epithelial cells from IBD patients. However, *Cela2* expression was unchanged after antibiotics treatment for 17 days in mice (analysis of GEO data base number GSE22648^29^), suggesting that microbiota changes are not sufficient to modify *Cela2a* expression.

We showed that ELA2A hyperactivity released at the apical side of epithelium triggers a decreased amount of membrane occludin and E-cadherin. This mechanism may lead to increased paracellular permeability to macromolecules and highlights the critical role of epithelium-derived proteases in the loss of barrier function.^30^ The observed reduction of occludin and E-cadherin presence in ELA2A transgenic mouse colonic tissues was not the consequence of displacement of protein into the cytoplasm (see immunohistochemistry data, Fig. 5b, d), neither was it the consequence of a reduced gene expression (Supplementary Fig. 6). Our data suggest that these proteins are degraded at the membrane level. Interestingly, epithelial intercellular junction proteins are differentially regulated in non-inflamed areas of intestinal mucosa from IBD patients.^24^ Our observation that ELA2A is also overexpressed in such areas could suggest that ELA2A participates to this intercellular junction protein dysregulation in non-inflamed IBD tissues.

Disruption of epithelial cell junction is also accompanied by a decreased expression of *TFF-3*, which is an important protein for recovery and maintenance of intestinal barrier function.^31^ In addition, histological analysis revealed goblet cell depletion in colonic crypts of hELA2-transgenic mice. Dysregulation of all these actors involved in barrier function could contribute to bacterial translocation observed in hELA2-transgenic mice. Nonetheless, the increase of *Reg3* transcriptional amounts could reflect a host response to a direct contact between the epithelium and luminal bacteria. Taken together, our results clearly demonstrate a major imprint of ELA2A overexpression on intestinal barrier breakdown.

In addition to an effect on intestinal barrier integrity, ELA2A hyperactivity also induced changes in chemokine/cytokine profiles *in cellulo* and in vivo. This is illustrated by *CXCL8* (in human) and *KC* (in mice) upregulation, but also by the decreased expression of *TGFβ, IL-6*, and *IL-10*, the latter being particularly important in intestinal homoeostasis. *TNFα* expression, a cytokine implicated in TJ disassembly, remained unchanged, further supporting the hypothesis that TJ changes observed herein were induced by elastase-mediated degradation of target protein.^32,33^ This is reinforced by the rapid deleterious effect of active ELA2A on epithelial barrier function observed after its addition to the apical side of Caco2 epithelial layers. Among cytokines and growth factors measured, *CXCL8* upregulation was a rapid and massive output in response to ELA2A hyperactivity in the extracellular environment of epithelial cells. We showed that ELA2 can directly send specific intracellular signals inducing *CXCL8* upregulation through the cleavage of PAR1. We also demonstrated that NFκB activation was involved in the ELA2A-induced upregulation of *CXCL8*.

The amount of *TGFβ* transcript was also decreased in ELA2A transgenic mice. This cytokine is a key anti-inflammatory signal that counteracts the pro-inflammatory effects of TNF*α*, IFN, and IL-1*β*,^34^ and considered as a protective signal in IBD.^35–37^ Although IL-6 is considered generally as a pro-inflammatory cytokine,^38^ recent reports highlighted a role for IL-6 in epithelial repair.^39,40^ The downregulation of *IL-6* in hELA2A-expressing mice would therefore support an impaired capacity for epithelial repair upon ELA2A hyperactivity. The ELA2A hyperactivity induced downregulation of *IL-10* mRNA amount observed in vivo further confirmed the pro-inflammatory phenotype of ELA2A epithelial overload. Together, these data provide evidence that ELA2A upregulation in intestinal epithelium turns on intestinal mucosal immunity by releasing the neutrophil chemoattractant CXCL8, and at the same time turns off tissue-healing processes leading to the recruitment of immune cells such as neutrophils and macrophages. This further suggests the implication of epithelial ELA2A in the amplification loop of tissue damage associated with IBD.

Human and mouse ELA2A are enzymatically similar, with 84% identity between both proteins. For this reason, we choose to overexpress the human enzyme in mice transgenic for epithelial ELA2A, with the hope that such mice could be used in the future for screening the efficacy of ELA2A inhibitors against the human enzyme in an in vivo model. Indeed, in vivo data presented here placed ELA2A signal upstream from a number of IBD-associated features. ELA2A epithelial overexpression was sufficient to induce in vivo barrier defects, cytokines/chemokine unbalance, and overall a pro-inflammatory phenotype. Precisely because ELA2A appears as a trigger of both barrier and immune balance defects, specific inhibition of this protease, using ELA2A selective inhibitors, could be considered as an interesting therapeutic approach to restore mucosal homoeostasis in IBD.

In conclusion, our results demonstrate that hyperactive ELA2A from IEC participates to key features of IBD pathogenesis: (i) increased permeability of intestinal epithelial barrier, which leads to penetration of luminal products into the mucosa and (ii) abnormal immune status of epithelial cells leading to chemokine-driven inflammation. These findings highlight possible novel therapeutic approaches targeting ELA2A. Such approaches would help restoring mucosal integrity, reinforcing current treatments aiming at prolonging remission phases in IBD patients.

## Materials and methods

### Patients

Human colonic tissues were obtained from individuals treated at the Centre Hospitalier de Toulouse (France). Biopsies were collected during colonoscopy procedures aimed at clinically evaluating the disease of established and well-characterized CD and UC patients or they were realized in individuals undergoing colon cancer screening who were otherwise healthy (here, healthy controls). The written and verbal informed consent was obtained before enrolment in the study, and the Ethics Committee approved the human research protocol (ClinicalTrials.gov identifier: NCT01990716 or DC-2015-2443). Fresh biopsies were proceeded as previously described^13^ and used for in situ zymography (*n* = 15 for healthy controls, *n* = 7 for CD patients, and *n* = 11 for UC patients) and immunostaining (*n* = 7 for healthy controls, *n* = 9 for CD, and *n* = 8 for UC). Human epithelial cells were isolated as previously described^41^ from colonic biopsies or surgical resections from patients suffering from colorectal cancer (control patients *n* = 16) or IBD (*n* = 12 CD, *n* = 10 UC). Tissues were harvested from non-macroscopically inflamed zones during colonoscopy or at the margin of the resection and at least 10 cm away from the tumour. The median of age is 57.03 years for controls (*n* = 33), 30.27 years for patients suffering from Crohn’s (*n* = 28), and 40.93 years for patients with UC (*n* = 23). The severity index of the disease is 155.9 (CDAI) for CD patients and 4.8 (Lichtiger index) for UC patients.

### In situ zymography

Frozen optimal cutting temperature (OCT) sections of colonic tissues from patients and mice (8-µm thickness) were rinsed with a washing solution (2% Tween-20) and incubated at 37 °C for 6 h with BODIPY-FL-Elastin (0.5 mM) by the EnzChek Elastase Assay Kit (Invitrogen) according to a previously published protocol.^18^ As control of elastase activity, slices were pre-treated with 100 nM ELAFIN for 15 min. All sections were analyzed with Zen 2009 software (Carl Zeiss), by observers unaware of the origin and treatments. Fluorescent intensity in epithelium was quantified in at least five crypts per slide using Image J software. For each experiment, mean of fluorescent intensity from healthy controls was used as a reference to normalize data throughout the different acquisitions.

### Immunostaining

Cryosections of 5 µm or transwell filter with epithelial cells were fixed with acetone 100%. Primary antibodies were incubated overnight at 4 °C in blocking buffer (1% BSA in PBS for 30 min). Following antibody anti-ELA2 (1/200; from Sigma-Aldrich for rabbit isoform or Santa Cruz for goat isoform), anti-ELAFIN (1/500; Santa Cruz Biotechnology), anti-EpCAM (1/200 dilution; from Abcam), anti-OCCLUDIN (1/250 dilution, from Invitrogen), and anti-E-cadherin (1/500; from Cell Signalling Technology) were used for immunohistochemistry. Anti-mouse, anti-rabbit, or anti-goat coupled with Alexa Fluor 555- and anti-mouse Alexa Fluor 488-conjugated secondary antibodies (1:1000 dilution; all from Invitrogen) were used. Slides were mounted and nuclei were stained with DAPI (4′,6-diamidino-2-phenylindole) fluorescent mounting medium (Invitrogen) and analyzed on a confocal microscope (Zeiss LSM710, Carl Zeiss) by observers unaware of treatments. To validate the absence of non-specific binding of anti-ELA2 antibodies, two different sources of anti-ELA2 (from rabbit, from goat) were used and, isotype controls were used, incubated at the same concentration and under the same experimental conditions. Images representative of each group were selected. Epcam staining was used to estimate the epithelium surface on each cryosections of biopsy. For each patient, eight random images were obtained, intensity of ELA2A staining and epithelium surface were quantified using image J software.

### Cell culture

Human colonic epithelial (Caco-2) cells (American Type Culture Collection, Manassas, VA) were grown in GlutaMAX DMEM (Invitrogen, Cergy-Pontoise, France) as previously described.^13^ Cells were grown on culture-treated plates (Becton Dickinson, Sparks, MD) under standard conditions and then transferred on transwell filter units with semipermeable filter membranes (0.4 µm) pores (Corning, New York, US). Caco-2 cells (2.10^5^ cells/well) were cultured in monolayer in 12-well transwell plates (Corning Life Sciences) (0.9–1.12 cm^2^ surface area) until confluence. After 21 days of culture (reaching trans-epithelial resistance > 500 Ω × cm^2^), cells were rinsed with PBS solution and Opti-MEM medium (Life Science) was added into apical and basolateral chambers.

Caco-2 monolayers were treated with recombinant ELA2A at various concentrations in apical compartment for 4 h. As control, ELA2A was pre-treated for 15 min with ELAFIN (R&D) or chymostatin (Sigma) as elastase inhibitors.

To generate human colonic organoids, fresh colonic tissues from control patients (*n* = 13) were harvested and colon crypts were isolated and cultured in a three-dimensional Matrigel matrix for 10–12 days as previously described.^42^ Organoids were generated in 48 wells in Matrigel plus culture medium (50% Wnt3a-conditioned medium (supernatant from L-Wnt3a cells), 50% advanced DMEM/F12, 100× GlutaMAX-CTS, 100× HEPES, serum-free B27, N2, N-acetylcysteine, nicotinamide, recombinant human epithelial growth factor, human Noggin, human R-spondin, gastrin, SB202190, LY2157299, and PGE2. The culture medium was changed every 2–3 days (without NAC and LY2157599). Organoids were passed once and were cultured as previously described for 11 days. Organoids were extracted from the Matrigel using the cell recovery solution (Corning) during 30 min, organoids were centrifuged, and then splitted by several up and down pipetting. About 800 organoids (100 organoids per well) were stimulated in culture medium without Matrigel by TLR ligands: ultrapure Pam(3)CSK(4) (TLR2/1, 500 ng/ml), HKLM (TLR2, 10^8^ cells/ml), FSL-1 (TLR2/6, 100 ng/ml), LPS (TLR4, 5 μg/ml), and flagellin (TLR5, 100 ng/ml) (InvivoGen) and then cultured for 24 h. Alternatively organoids were pre-treated with inhibitors Bay 11- 7082 (10 μM), GB 88 (5 μM), and F16357 (5 μM) for 45 min prior addition of ELA2 (0.25 mU/ml) and then cultured for 8 h in Opti-MEM.

### RT-PCR analysis

RNA was isolated using the RNeasy Kit (Qiagen). One to five micrograms of RNA was reverse transcribed following the recommendations of the Superscript first strand synthesis system. PCR was performed with GoTaq polymerase (Promega), using 1/10 to undiluted cDNA as a matrix, with primers indicated in Supplementary Table 1. When needed, amplicons were cloned into pGEM-T easy (Promega) and then sequenced using BigDye Terminator.

Levels of specific mRNA transcripts were determined using SYBR Green PCR Master Mix and qPCR was performed on a LightCycler 480 (Roche Diagnostics) using primers described in Supplementary Table 1. Relative expression of targeted genes was compared to hypoxanthine phosphoribosyltransferase or glyceraldehyde-3-phosphate dehydrogenase expression.

### Production and purification of recombinant human ELA2A

Human form of *CELA2A* cDNA was subcloned in pCI-neo vector (Promega) with HA-tag in fusion in C-term of encoding ELA2A sequence. This plasmid was transfected into CHO-Ki cells (ATCC^®^ CCL-61™) using GeneJuice transfection reagent according to the manufacturer’s instructions (Merck). The cells were selected with G418 (Invitrogen SARL) at 1.6 mg .ml^−1^ during 10 days in medium HAMF12, 10% SVF, non-essential amino acid. The cells were grown in medium with G418 (0.8 mg .ml^−1^). Before harvesting medium, cells were washed with PBS five times and replaced by Opti-MEM (Invitrogen SARL). Opti-MEM medium was collected 48 h later containing pro-ELA2A_HA_. ELA2A_HA_ was purified using affinity column for HA epitope following manufacturer’s instruction (Supplementary Fig. 3c). Heterologous protein was eluted using 3M NaSCN and pH was immediately neutralized. Pro-ELA2A_HA_ was activated with trypsin–agarose (Thermo Scientific™ Pierce™) for 90 min at 37 °C. Trypsin activity released from agarose was blocked using activity-based probe coupled with biotin^16^ and eliminated using streptavidin resin following manufacturer’s instructions (GE Healthcare Life Science). After centrifugation, active ELA2A_HA_ was collected into the supernatant and elastolytic activity was quantified.

Elastolytic activity was measured with Ala-Ala-Pro-Met-AMC (0.75 mM, Bachem) or Elastin-FITC (5 μg/ml, Invitrogen) as substrate. Samples were resuspended in buffer (100 mM Tris-HCl, 1 mM CaCl_2_, 0.1% BSA, 1% Chaps). The change in fluorescence was measured over 30 min at 37 °C on a microplate reader NOVOstar (BMG Labtech) to calculate the rate of substrate degradation.

For activation assay with different forms of TRYPSINS, equal specific enzymatic activity for PRSS3 (R&D) and PRSS1-2 mix (Sigma) quantified with the Gly-Pro-Arg-AMC (50 μM, Bachem) substrate was added in each well. Pro-ELA2 at 0.3 μM was incubated with 0.054 μM active trypsin for 60 min at 37 °C in 100 mM Tris-HCl, 1 mM CaCl_2_, pH 8. The Ala-Ala-Pro-Val-AMC substrate degradation by activated ELA2A was followed over time using 1/5 reaction volume in 100 mM Tris-HCl, 1 mM CaCl_2_, 50 mM Cacl_2_, 1% Chaps, pH 7.5.

### Western blot

Cultured cells were crushed in a protein extraction buffer containing 50 mM Tris-HCl, pH 8.0, 120 mM NaCl, 5 mM EDTA, 0.5% Nonidet-P40, and 1X Protease Inhibitor Cocktail Tablets (EDTA-free; Roche) with Ultra-Turrax. Total lysates were sonicated 3× for 5 s and were clarified from insoluble material by centrifugation at 12,000 × *g*, 4 °C for 30 min.

Cellular fractions (soluble and insoluble) were obtained by cellular lysis in 1% Triton X-100, 100 mM NaCl, 10 mM HEPES pH 7.6, 1 mM EDTA and 1X Protease Inhibitor Cocktail Tablets (EDTA-free; Roche) and were prepared according to Jacob et al.^43^ Briefly, lysates were centrifuged at 15,000 × *g*, 4 °C for 30 min, and the supernatant formed the soluble fraction. The pellet was sonicated in Triton buffer containing 1% SDS and centrifuged at 15,000 × *g*, 4 °C for 5 min, to obtain the insoluble fraction corresponding to an enriched membrane fraction.

Protein content was quantified and equal amounts (50 µg) were separated by SDS–polyacrylamide gel electrophoresis (7–15%) and transferred to nitrocellulose membrane (Whatman, VWR International). Membranes were blocked in Tris-buffered saline with 5% non-fat dry milk and 1% BSA. Blots were incubated with a primary antibody overnight at 4 °C. The following primary antibodies were used: rabbit (1:500 dilution) or goat anti-ELA2 (dilution 1:200), rabbit anti-OCCLUDIN (1:500 dilution), E-cadherin (1:1000 dilution), mouse monoclonal anti-β-actin (1:10000 dilution; from Sigma-Aldrich), and goat anti-VINCULIN (1:5000 dilution; from Sigma-Aldrich). Mouse, rabbit, or goat horseradish peroxidase-conjugated secondary antibodies (1:3000 dilution; from Cell Signalling Technology) were used for incubation for 1 h at room temperature and then blots were visualized by chemiluminescence on ChemiDoc (Bio-Rad). Signal density was calculated using Image Lab software (Bio-Rad). Actin was used as a loading control for total and soluble fraction and Vinculin as control for insoluble fraction.

### In vitro permeability measurements

To assess epithelial permeability, FITC–dextran (4 kDa, 100 μM) was added to the apical compartment of the transwells for 4 h. Basolateral samplings were harvested at each sample timepoint. The apical-to-basolateral flux of FITC–dextran was measured on a fluorescence plate reader (NOVOstar, BMG Labtech) with an excitation wavelength of 496 nm, emission at 524 nm, and a cut off of 515 nm. Paracellular permeability to dextran–fluorescein isothiocyanate was expressed in relative permeability or µmole × cm^2^ of dextran–fluorescein isothiocyanate. The synthetic molecules F16357,^44^ GB88, and HC047067 corresponding to PAR1, PAR2, or TRPV4 antagonist, respectively, were pre-incubated for 45 min at 5 μM or 10 μM for HC047067.

### Animal models

The Animal Care and Ethics Committee of US006/CREFE (2016112509278235v2) approved all experimental procedures and they were performed following the guide for the care and use of laboratory animals of the European Council. All animals were maintained under 12-h light–dark cycles with free access to food and water, except for being fasted 4 h before the dextran–FITC per os administration.

Germ-free mice were obtained by two stages embryo transfer and housed in flexible film gnotobiotic isolators at the Axenic Gnotobiotic Facility of McMaster (Ontario, Canada).

Colonic inflammation was induced on WT C57BL/6 8-week-old mice (Janvier) by treatment with DSS (MP Bio- medicals) dissolved in drinking water (5%, w/v) for 7 days.^18^

A knock-in mouse line was generated to allow conditional expression of ELA2A Flag-tagged and EYFP cDNAs under the control of CMV early enhancer/chicken β actin (CAG) promoter at permissive Rosa26 locus (Supplementary Fig. 3) (Charles River). Between the promoter and the transgene, a floxed STOP cassette has been included which efficiently terminates transcription. Interbreeding the mouse line with Cre-expressing mice^45^ and tamoxifen induction leads to the excision of the floxed STOP cassette and the activation of expression in IEC specifically. Littermates carrying *hCELA2A* floxed cassette but not the *pVillin::Cre-ER*^*T2*^ transgene (Cre^−^ transgenic *hCELA2A* mice) served as control mice for the described experiments. Control (Cre^−^ transgenic *hCELA2A* mice) and Tg-hELA2A (*pVillin::Cre-ER*^*T2*^ *hCELA2*) were used with heterozygous genotype.

#### Tamoxifen induction

Mice of >7 weeks were injected intraperitoneally with tamoxifen (0.2 mg/animal dissolved in sterile corn oil, Sigma) once daily for 5 days, and once every 2 days for additional 3 weeks. Both groups (control and *pVillin::Cre-ER*^*T2*^ hCELA2) were subjected to tamoxifen injection. In vivo intestinal permeability was assessed by quantification of FITC–dextran (100 mg/kg body weight) from serum as previously described.^46^ After sacrifice, colons were harvested for measurement of inflammation parameters macroscopic damage score, bowel thickness, and elastolytic activity as previously described.^12,13^ Pieces of tissue were collected and kept in OCT compound, paraffin, or trizol for further analysis. To enrich the samples in epithelial cells, the luminal side of surface of colon was scratched with a microcopy glass slide. Overexpression of *hCELA2A* was confirmed by RT-PCR, western blot for Flag-tag (1/400, Sigma), and immunostaining.

### Statistical analysis

Statistical analysis was performed with GraphPad Prism version 5.0. According to dataset structure, multiple comparisons were performed using either Student’s *t* test, Bonferroni post-hoc test, or Mann−Whitney post-hoc test following ANOVA analysis, as indicated in figures legends. *p* values < 0.05 were considered to be statistically significant (*p < 0.05, **p < 0.01, and ***p < 0.001). Figures using human biological samples are represented with scatter plot with the median. Statistical analysis was processed only when number of patients was superior to five. Unless otherwise stated, all other graphics are expressed as mean ± SEM or scatter plot with mean. Independent biological replicates as well as the total *n* number values are indicated in figures legends.

## Supplementary information

Supplementary Data
