# Supplementary material for: Epithelial production of elastase is increased in inflammatory bowel disease and causes mucosal inflammation
Source: Mucosal Immunol. 2021 Mar 5;14(3):667–78. doi: 10.1038/s41385-021-00375-w (PMC8075934; doi:10.1038/s41385-021-00375-w)
Supplement: Supplementary file 1 — Supplementary Data [file 41385_2021_375_MOESM1_ESM.pdf]

Supplementary Data

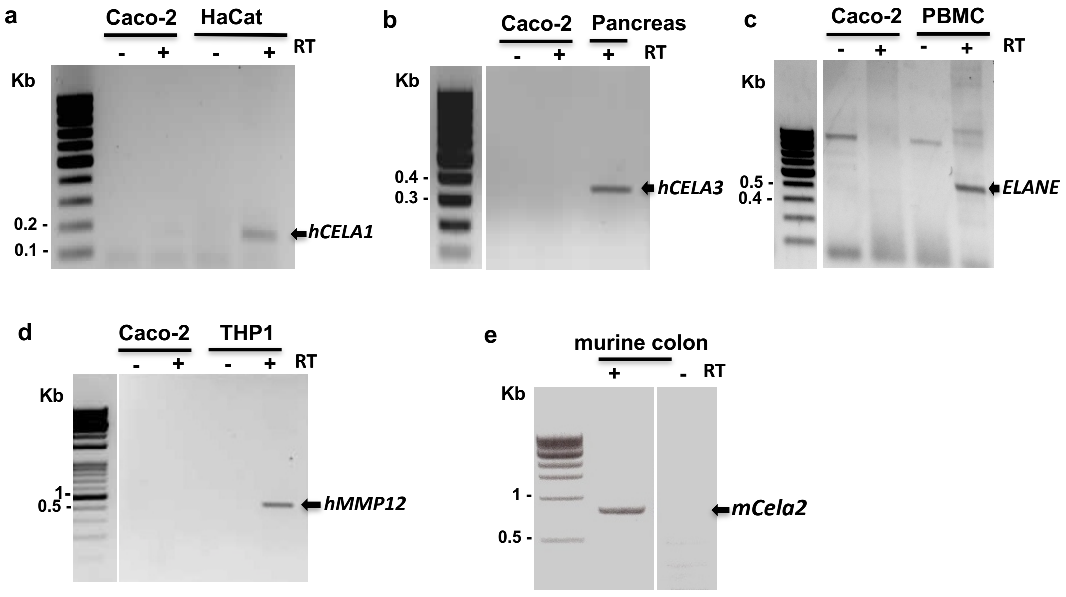

Supplementary Fig. 1: Expression screening for proteases exhibiting elastase activity . RT-PCR was performed to amplify the human *CELA1* **(a),** *CELA3* (**b),** *ELANE* **(c)** and *MMP12* **(d)** cDNA from Caco2 transcripts. mRNA from human keratinocyte cell line (HaCat) was used as positive control for *CELA1*, from human pancreas for *CELA3*, from peripheral blood monononuclear cell (PBMC) for *ELANE* expression and human macrophage cell line (THP1) to detect *MMP12* cDNA. **e** RT-PCR for *Cela2A* using mRNA from murine colon. Refer to Supplementary Table 1 for primer sequences.

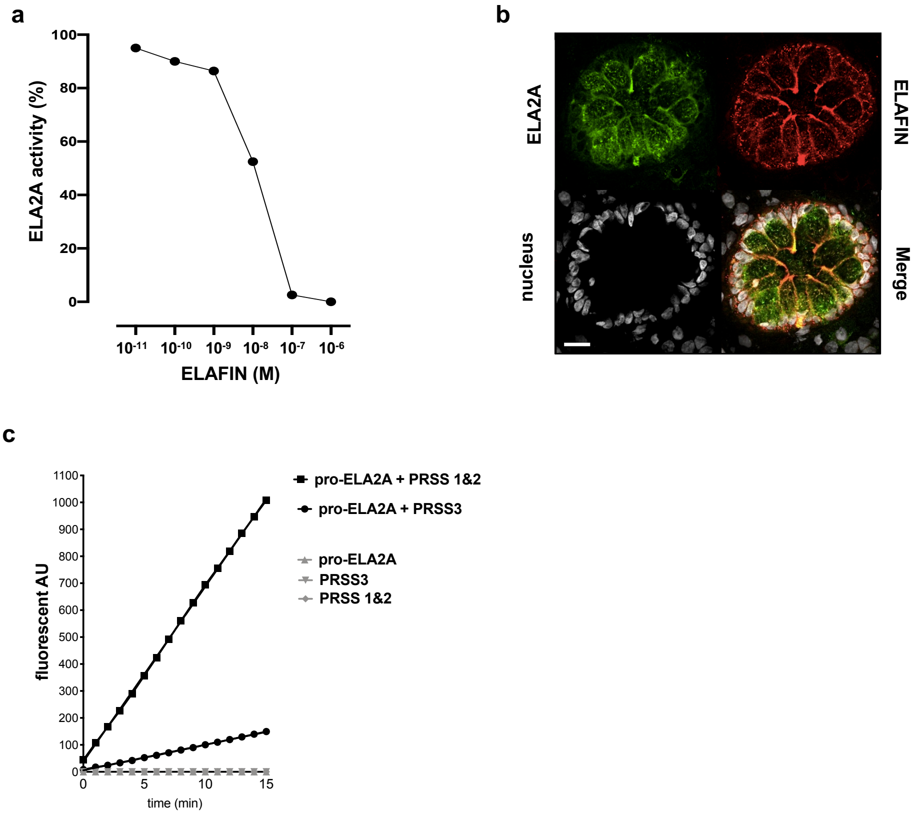

Supplementary Fig. 2: Regulators of ELA2 activity

a ELAFIN capacity to inhibit active ELA2A. ELA2A was incubated with increased concentrations of ELAFIN and then the Ala-Ala-Pro-Val-AMC substrate degradation by active ELA2A was followed over time. The curve represents the percentage of resulting proteolytic activity. Graph is representative of 3 independent experiments. **b** Immunostaining of ELA2A (green) and ELAFIN (red) on cross-section of healthy human colon. Nuclei are detected by DAPI staining and appear in blue. Scale bar = 20 µm. Images are representative of n=3 healthy controls. **c** Activation of pro-ELA2A by PRSS3 and PRSS1-2 mix (Sigma). PRSS isoforms were added to pro-ELA2A in a concentration ratio of 0.05/1 and pre-incubated for 30 min before adding the substrate. Pro-ELA2A displayed no activity as well as PRSS1-2 and 3. Graph is representative of 3 independent experiments.

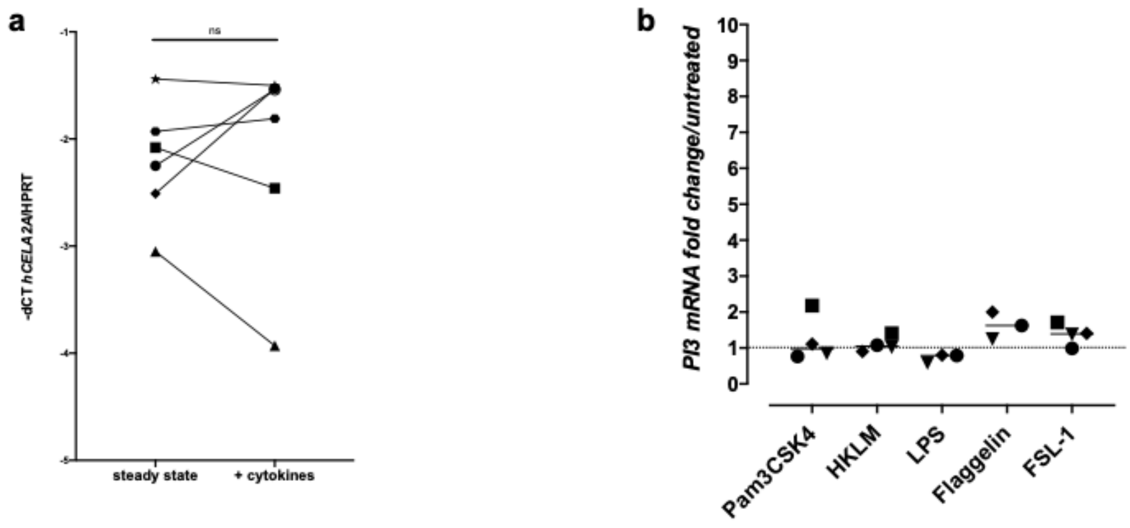

Supplementary Fig. 3

a Human colon organoids from 6 control patients were treated by a inflammatory cytokine cocktail for 7 days (IL-1β, IL6, TNF-α (PeproTech) at the concentration of 10 ng/ml). *CELA2A* gene expression was quantified. A statistical analysis based on a Dunn's post-hoc test after a Friedmann test was used. **b** Transcriptional level of gene encoding ELAFIN (*PI3*) was determined in human colon organoid after stimulation for 24h with TLR ligands.

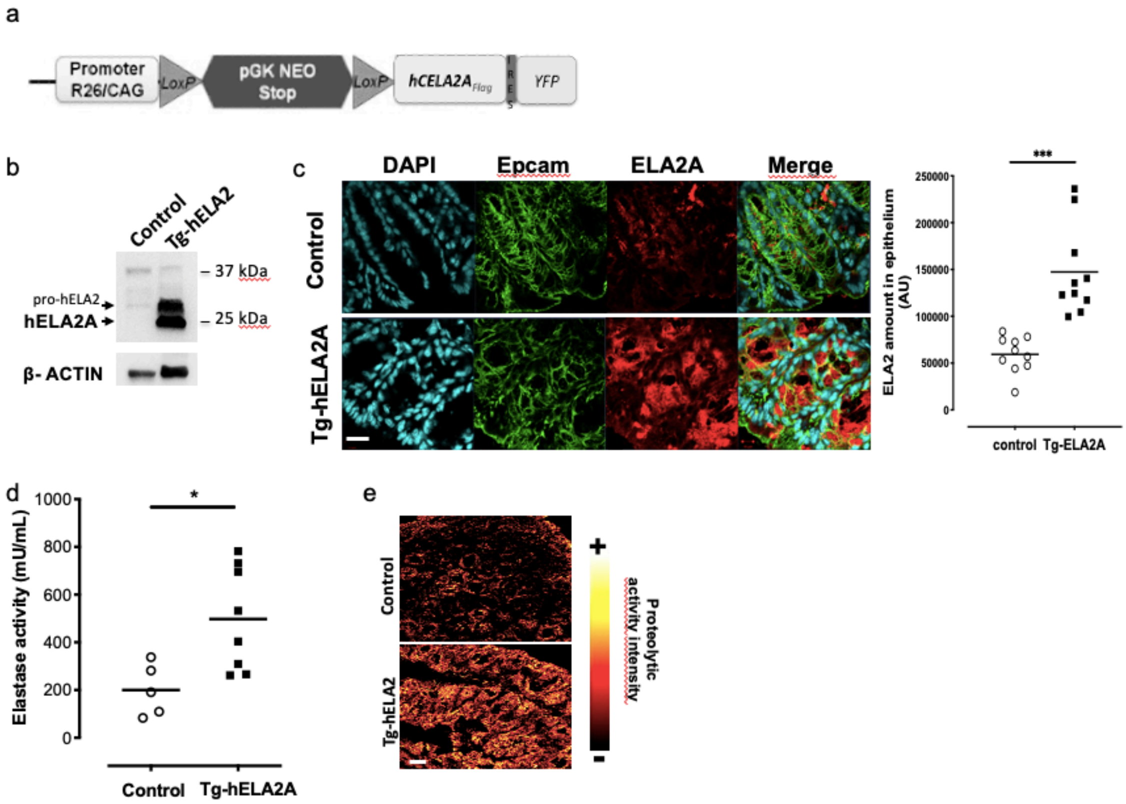

Supplementary Fig. 4: Generation and characterization of mice overexpressing the human full-length CELA2A gene in intestinal epithelial cells.

a Schematic structure of the transgene. The human full-length CELA2A cDNA was fused at its 3’ end with the FLAG epitope and cloned downstream of the floxed Neo stop cassette. An IRES motif sequence followed by the eYFP protein marker was cloned in fusion with the *hCELA2A* cDNA. **b** *hCELA2A* over-expression was confirmed by Western blot analysis using anti-Flag antibody on colonic mucosal extract. **c** ELA2 immunostaining (red) on colonic tissue from mice. Specific epithelial cell marker, Epcam, staining is represented in green. Nuclei are detected by DAPI staining and appear in blue. Scale bar = 20 µm. **d** Elastolytic activity in colonic washes from control or ELA2A transgenic mice is expressed as mU of elastase per ml. Data are represented as dots and means are indicated as lines. * $p<0.05$ compared to control mice using two tailed t-test with Mann-Whitney post-test. **e** *In situ* zymography of elastolytic activity in colon from mice. Scale bar = 20 um.

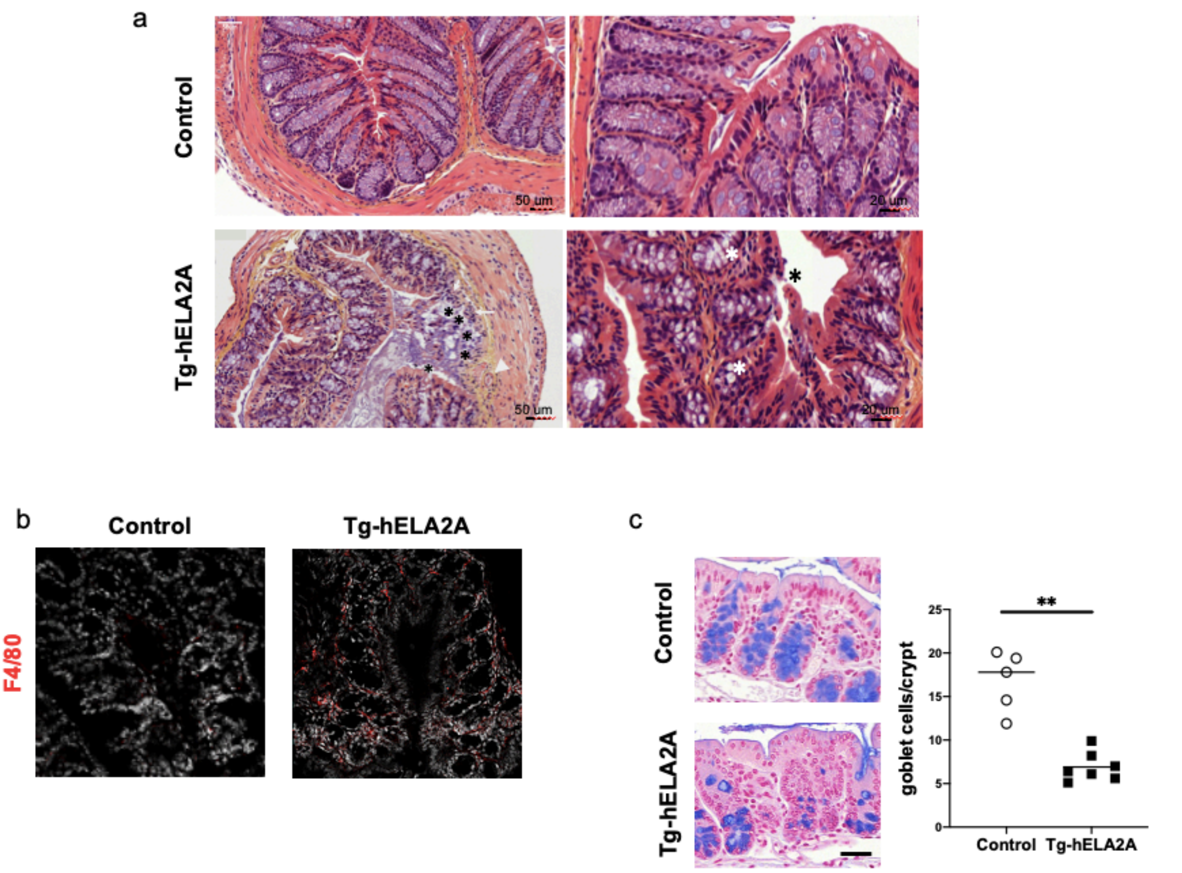

Supplementary Fig. 5: Histological analysis of colon from hELA2-transgenic mise

a Histology images of colon tissues from controls and ELA2A-transgenic mice displaying multiple erosions of the mucosa (black stars), where the epithelium was largely damaged or even absent, infiltrated mononuclear cells (white arrow), and vasodilated blood vessels (arrow heads) in ELA2A transgenic mice. In tissues from wild-type mice (controls left panels), goblet cells were clearly filled with mucus, while in ELA2A-transgenic mice, goblet cell depletion was frequently observed (white stars). **b** F4/80 Immunostaining in controls and ELA2A-transgenic mice displaying large influx of macrophages in ELA2A transgenic mice. scale bar XX µm **c** Goblet cell-associated mucus was quantified from Alcian blue stained sections, scale bar 50 µm. Data represent mean of number of goblet cells per crypt in control and ELA2 transgenic mice (10 crypt par animal, n= 5-7 mice per group).

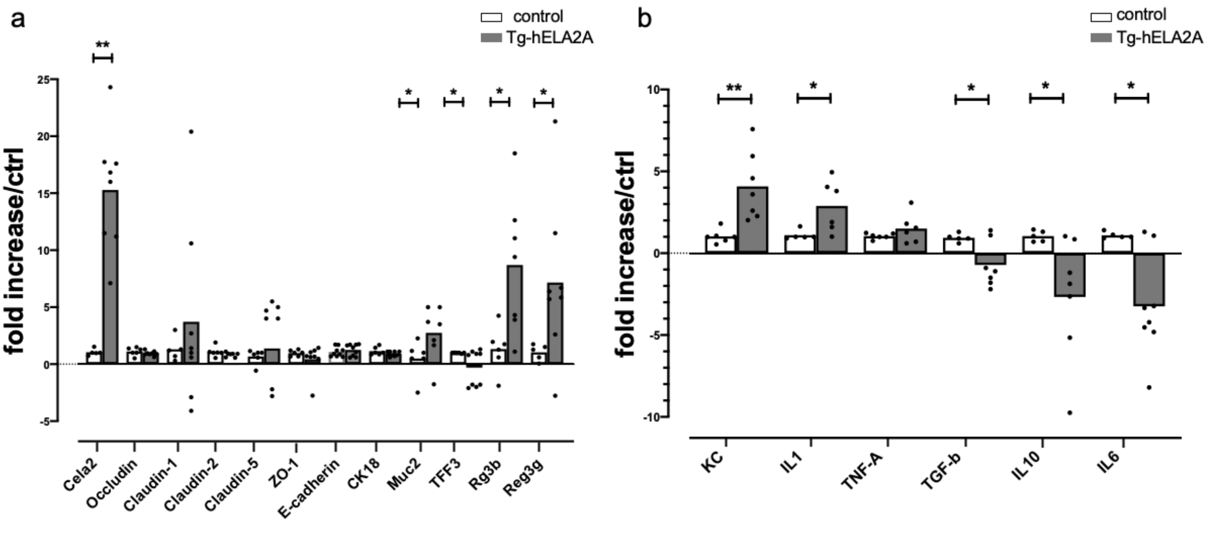

Supplementary Fig. 6: Profile of gene expression in colonic mucosae and whole colon tissue from Tg-hELA2A mice.

a Relative gene expression of epithelial cell markers quantified in mucosal extract from mouse colon of hELA2A transgenic mice compared to controls. Pooled data are from 6-8 mice per group. **b** Relative cytokine gene expression in colonic tissues of hELA2A transgenic mice compared to controls. Pooled data are from 6-8 mice per group. * $p<0.05$, ** $p<0.01$ and *** $p< 0.001$ compared to control mice using multiple t-test with Mann-Whitney post-test.

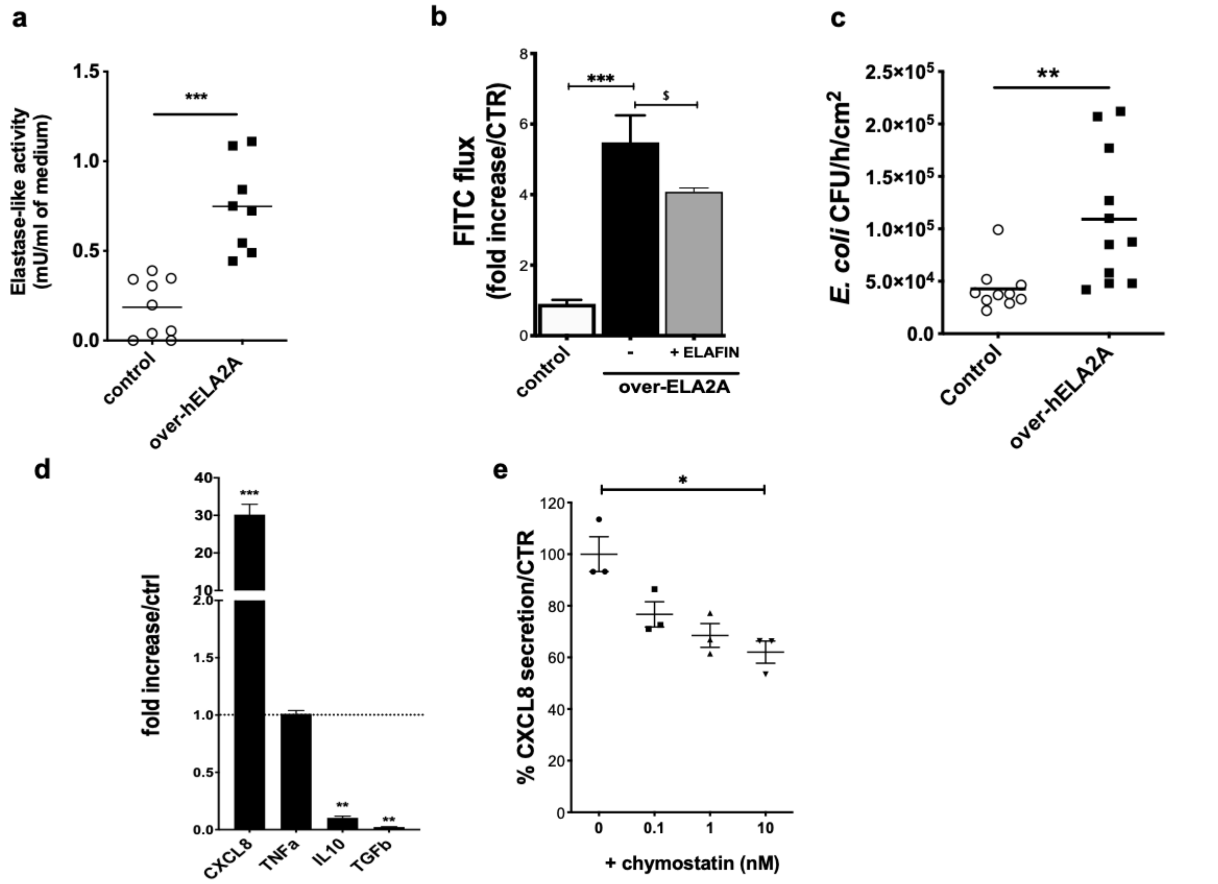

Supplementary Fig. 7: Increased extracellular ELA2A in Caco-2 cells alters barrier function and cytokine expression profile

a Elastolytic activity into medium from ELA2A-overerexpressing Caco-2 cells (over-ELA2A). Caco-2 cells were genetically modified, an expression cassette was introduced in genomic DNA to allow the constitutive expression of *hCELA2A* under the pCMV promoter. Controls cells correspond to the Caco-2 cells genetically modified by the empty expression cassette. Data are represented as dots and means are indicated as lines. *p<0.05 using two-tailed T-test. **b** Paracellular permeability measurements in control *versus* ELA2A-overexpressing (over-ELA2A) Caco-2 cell monolayers. ELAFIN (10nM) was added 12H hours prior to evaluate barrier function of over-ELA2A monolayer. The graph indicates the fold increase in 4 kDa dextran flux of over-ELA2A cells compared to control cells. *** p<0.001 compared to control condition, $ p<0.01 compared to over-ELA2A cells using Kruskall wallis test corrected with a Dunn's multiple comparison test (n=4 to 16 per group). **c** Bacterial translocation across control and ELA2A-overexpressing Caco-2 monolayers was assessed with heat killed fluorescent-conjugated *E. coli* K12 strain (n=12 per group). ** p<0.01 using two tailed t-test with Mann-

Whitney post-test. **d** Cytokine gene expression was quantified by RT-qPCR in ELA2A-overexpressing Caco-2 cells. The graph indicates the fold increase of the different mRNAs encoding cytokines in overexpressing Caco-2 cells *versus* control cells (dashed line). Data are expressed as means ± SEM of n=3 for each group in 3 independent experiments, **$p<0.01$, ***$p< 0.001$ compared to control using two tailed t-test. **e** Presence of chymostatin in culture medium of e over-expressing ELA2A cells reduces in a dose-dependent manner the release of CXCL8. * $p<0.05$ compared to untreated ELA2 overexpressing Caco-2 using a Dunn's post-hoc test.

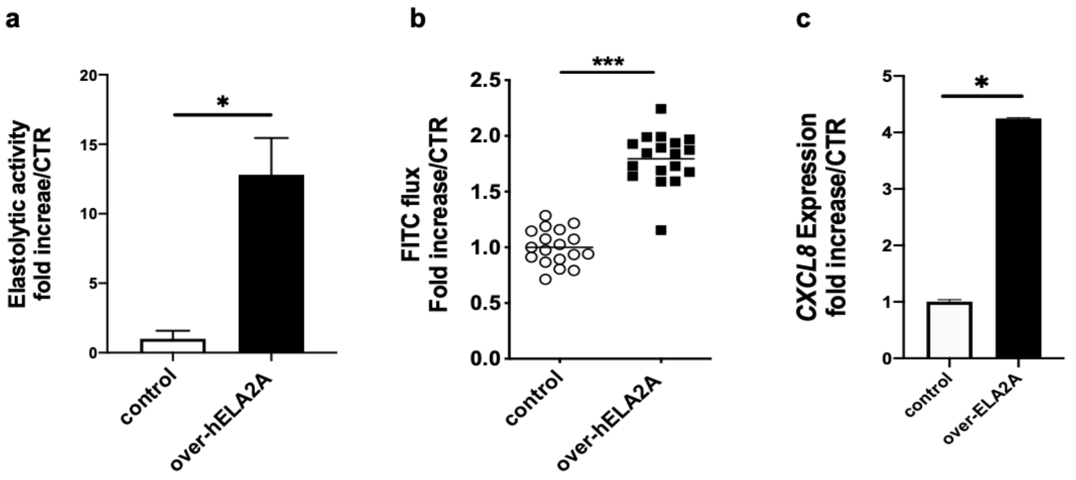

Supplementary Fig. 8: HT-29 epithelial cells overexpressing ELA2 display loss of barrier function and increase of pro-inflammatory CXCL8 cytokine.

a Elastolytic activity into medium from ELA2A-overexpressing HT-29 cells (over-hELA2A). Data are represented as means ± SEM of n=3 for each group in 3 independent experiments. *p<0.05 using two-tailed T-test. **b** Paracellular permeability measurements in control *versus* ELA2A-overexpressing (over-ELA2A) HT-29 cell monolayers. The graph indicates the fold increase in 4 kDa dextran flux of Tg-ELA2A cells compared to control cells. *** p<0.001 for all comparison using two tailed t-test with Mann-Whitney post-test (n=20 per group). **c** *CXCL8* gene expression in ELA2A-overexpressing HT-29 cells. The graph indicates the fold increase of CXCL8 mRNA amount in ELA2A-overexpressing HT-29 cells *versus* control cells. Data are expressed as means ± SEM of n=3 for each group in 2 independent experiments, **p<0.01, ***p< 0.001 compared to control using two tailed t-test.

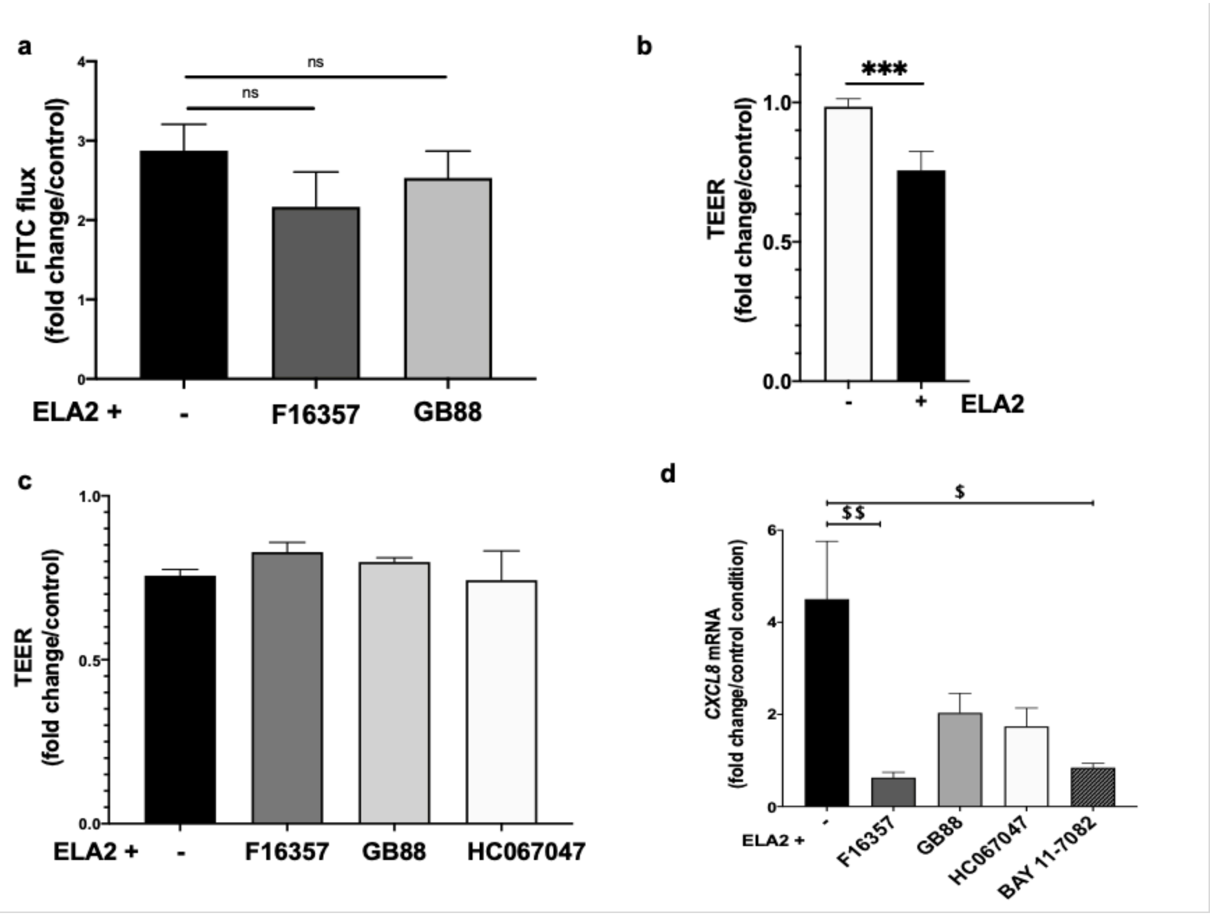

Supplementary Fig. 9: Effect of PARs and TRPV4 antagonists on leaky pathway permeability and CXCL8 up-regulation induced by ELA2 activity.

a Antagonist of Protease Activated Receptor-1 (PAR1, F16357 at 5 uM), of PAR2 (GB88 at 5 uM) or of TRPV4 (HC067047 at 10 uM) was added on the apical side of the Caco-2 monolayer cultured on transwell, 45 min prior to addition of ELA2 (20 mU/ml). FITC-dextran flux through the epithelial monolayer were measured 4H after. Data are expressed as means ± SEM of n=3 for each group in 3 independent experiments. **b, c** Trans-epithelial electrical resistance measurement of Caco-2 monolayer after 4H incubation with ELA2 (20 mU/ml) in apical medium (b). Antagonist of Protease Activated Receptor-1 (PAR1, F16357), of PAR2 (GB88) or of TRPV4 (HC067047) was added on the apical side of the Caco-2 monolayer cultured on transwell, 45 min prior to addition of ELA2 and TEER was then measured 4H after (c). Data are expressed as means ± SEM of n≥3 for each group in 3 independent experiments. **c**. Antagonist of PAR-1, -2, TRPV4 or NFkB inhibitor was added on the apical side of Caco-2 monolayer cultured on transwell 45 min prior to the ELA2 addition (50 mU/ml), and 8H latter mRNA were extracted to quantify *CXCL8* expression level. Data corresponding to the *CXCL8* gene expression after treatment compared to the control conditions (addition of chemical

compound alone) are expressed as means ± SEM of n=3-5, \$ $p<0.05$, \$\$ $p<0.01$ compared to ELA2 stimulation condition using Kruskall wallis test corrected with a Dunn's multiple comparison test

Primer sequences	Foward	Reverse
hCELA2A	ATGATAAGGACGCTGCTGCT	TTAGTTATTTGCAATCACCGAATTG
mCELA2	ATGATCAGGACACTGCTGC	TTAGTTCCTTGCCATCACC
hELANE	ACTGCGTGGCGAATGTAAACGTCC	AATTCCGTGGATTAGCCCGTTGCAG
hCELA1	GGCTGGAGACCATAACCTGA	CCTGGGTGAAGACTGTAGGC
hCELA3	TTTGTGCATCCACTCTGGAA	GCCTTGGTTCTAGTGGCTTG
hMMP12	ACACATTTCGCCTCTCTGCT	CCAGGGTCCATCTGTCT

Primer sequences for real-time PCR	Foward	Reverse
mZO-1	CGTTATGATCCAGCCCAG	GCTGGTTTACTCTGAGATGG
mClaudin-1	CCTACTTTCCTGCTCCTG	TGTCCATTTTGTATTTGCTCC -TCC
mClaudin2	CCACAGATACTTGTAAGGAG	CCAAAAGGCCTAGGATGTAG
mClaudin-5	CAATGGCGATTACGACAAG	GGCTAGTGATGGTCAACG
mOccludin	ACCCTGACCACTATGAAAC	CGTCTAGTTCTGCCTGTAAG
mE-Cadherin	TGACTCGAAATGATGTGGCTCCCA	CTGCCCTCGTAATCGAACACCAA
mMuc2	GTAAACTGCTCTCTGGACTG	CTTGGAAGACGTGGTAGATG
mCK18	TAACAGGAACTCCCGTGACA	GCAGCTTCTTGATCGTGGTG
mTFF3	CCTGGTTGCTGGGTCCTCTG	GCCACGGTTGTTACACTGCTC
mREG3b	CTGGTTTGATGCAGAACTG	TGTTACTCCATTCCCATCC
mREG3g	CGACACTGGGCTATGAAC	TCTCCACTTCAGAAATCCTG
KC	AGCCACACTCAAGAATGGTC	GTCAGAAGCCAGCGTTCAC
mIL1b	ACCTTCCAGGATGAGGACATGAG	CATCCCATGAGTCACAGAGGATG
mTNFα	TGGGAAGACAGGGAAAAC	GGCTGAAAGGATGGAAATG
mIL10	ATCGATTTCTCCCCTGTGAA	TGGCCTTGTAGACACCTTGG
mTGFb	GACCCCCACTGATACGCCT	GCTGAATCGAAAGCCCTGTA
mIL6	CAAAGCCAGAGTCCTTCAGAG	GCCACTCCTTCTGTGACTCC
mCELA2A	CTGGCACCATTCTCCCGAG	TTCCCATTGGTCTGCAGCA
hELA2	TTGTGGTGCACAAGGACTGG	GCAATGTCGTTCCCTTTGGAG
PI3	GCAGCTTCTTGATCGTGGTG	TAACAGGAACTCCCGTGACA
HPRT	TGG GAG GCC ATC ACA TTG T	TCC AGC AGG TCA GCA AAG AA

Supplementary Table 1. Primers used for PCR and quantitative RT-PCR studie
